# Ligand-Based Pharmacophore Modeling Using Novel 3D Pharmacophore Signatures

**DOI:** 10.3390/molecules23123094

**Published:** 2018-11-27

**Authors:** Alina Kutlushina, Aigul Khakimova, Timur Madzhidov, Pavel Polishchuk

**Affiliations:** 1 A.M. Butlerov Institute of Chemistry, Kazan Federal University, Kremlevskaya Str. 18, 420008 Kazan, Russia; Alina.Kutlushina@pharminnotech.com (A.K.); aigul03.14@gmail.com (A.K.); tmadzhidov@gmail.com (T.M.); 2 Institute of Molecular and Translational Medicine, Faculty of Medicine and Dentistry, Palacký University and University Hospital in Olomouc, Hnevotinska 5, 77900 Olomouc, Czech Republic

**Keywords:** 3D pharmacophore signatures, 3D pharmacophore hash, pharmacophore modeling, ligand-based modeling

## Abstract

Pharmacophore modeling is a widely used strategy for finding new hit molecules. Since not all protein targets have available 3D structures, ligand-based approaches are still useful. Currently, there are just a few free ligand-based pharmacophore modeling tools, and these have a lot of restrictions, e.g., using a template molecule for alignment. We developed a new approach to 3D pharmacophore representation and matching which does not require pharmacophore alignment. This representation can be used to quickly find identical pharmacophores in a given set. Based on this representation, a 3D pharmacophore ligand-based modeling approach to search for pharmacophores which preferably match active compounds and do not match inactive ones was developed. The approach searches for 3D pharmacophore models starting from 2D structures of available active and inactive compounds. The implemented approach was successfully applied for several retrospective studies. The results were compared to a 2D similarity search, demonstrating some of the advantages of the developed 3D pharmacophore models. Also, the generated 3D pharmacophore models were able to match the 3D poses of known ligands from their protein-ligand complexes, confirming the validity of the models. The developed approach is available as an open-source software tool: http://www.qsar4u.com/pages/pmapper.php and https://github.com/meddwl/psearch.

## 1. Introduction

Pharmacophore models have proved to be useful for the selection of focused sets of compounds [1,2,3,4]. There are two kinds of pharmacophores: (i) structure-based pharmacophores derived directly from X-ray structures of protein-ligand complexes, and (ii) ligand-based pharmacophores derived from structures of known active compounds. While the number of solved 3D protein structures increases each year there are still a lot of targets which are not available for structure-based modeling. Therefore, ligand-based modeling approaches remain attractive for researchers.

There are many ligand-based modeling tools, but almost all of them are commercial (LigandScout, Discovery Studio, MOE, PHASE, etc.) [5]. These programs use different algorithms for common pharmacophore identification based on genetic optimization [6,7,8], clique detection [9] and pharmacophore alignment with ranking [10,11]. PharmaGist is one of the known free tools for ligand-based pharmacophore generation [12]. To find features that are common for all active compounds, PharmaGist takes a pivot ligand in its “bioactive” conformation and aligns all other compounds to it. However, the “bioactive” conformations of active compounds are not always available, and this substantially limits program applicability. Another freely available tool is USRCAT, which is a shape recognition tool with pharmacophoric constraints [13]. This approach takes a given conformer of a query compound and performs screening. Therefore, it is not a true modeling approach, but rather a screening one. Pharmer is a free program which also performs fast virtual screening using given pharmacophore models [14]. Thus, there are no free tools which can derive 3D pharmacophore models from a given set of compounds without knowledge about their “bioactive” 3D structures. Moreover, many existing approaches for developing pharmacophore models utilize information only about active compounds. This can lead to less selective pharmacophore models, and usage of additional information about inactive compounds might be advantageous.

In this study, we developed a new approach for 3D pharmacophore representation and matching which does not require alignment of compounds/pharmacophores to find a common pharmacophore. It was tested on several retrospective case studies and compared to a 2D pharmacophore similarity search. It was also checked whether developed 3D ligand-based pharmacophore models can match the poses of available ligands from PDB protein-ligand complexes. The approach was implemented as a software tool which is available at http://www.qsar4u.com/pages/pmapper.php and https://github.com/meddwl/psearch.

## 2. Materials and Methods

### 2.1. Data Sets

Three data sets were used for the development of ligand-based pharmacophore models: inhibitors of acetylcholinestarase (AChE), inhibitors of cytochrome P450 3A4 (CYP450 3A4), and antagonists of adenosine 2a receptor (A2a). These targets were chosen because there was sufficient data about active and inactive compounds for model development, and there were many 3D protein-ligand complexes in the Protein Data Bank for validation of the obtained models. While these targets have high importance for the treatment of various conditions and diseases, the goal of this study was to develop and validate the new pharmacophore modeling approach, rather than develop models against particular targets.

All data sets were downloaded from the ChEMBL database. The structures of compounds were curated according to the workflow: https://bitbucket.imtm.cz/projects/STD/repos/std/browse. Compounds were categorized as active or inactive based on their respective IC_50_ values (Table 1). Data sets are provided in the Supplementary materials.

### 2.2. 3D Pharmacophore Signature Representation

**Figure 1:**
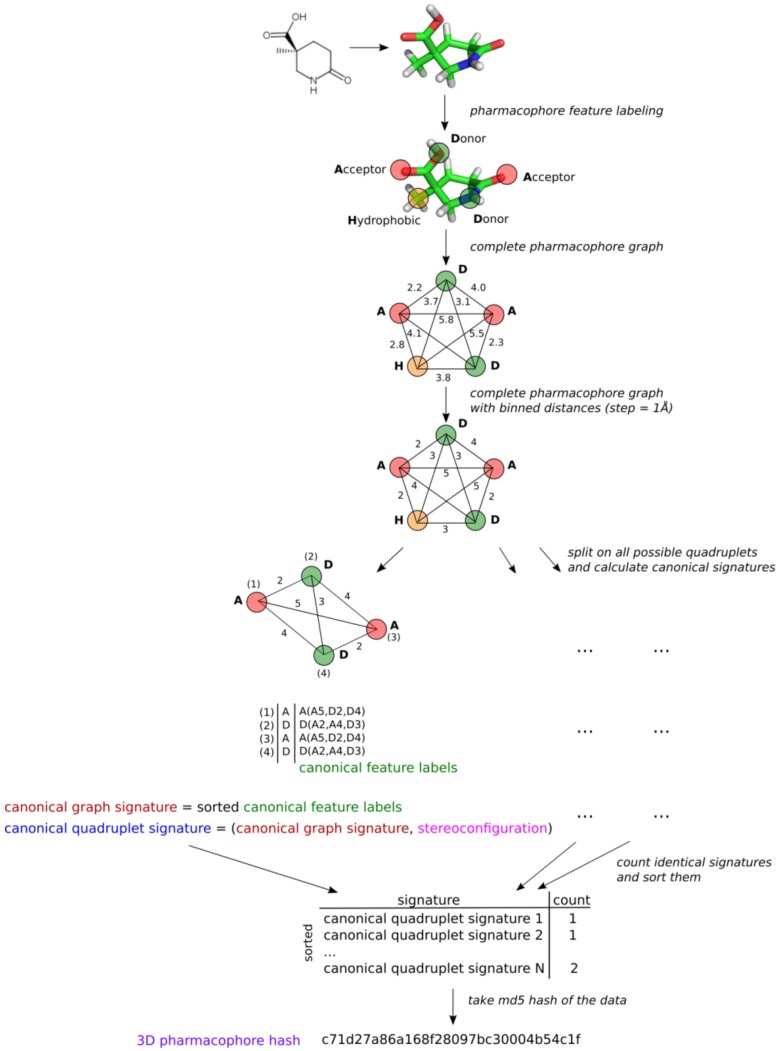
An example of the generation of a 3D pharmacophore hash.

The main principle of 3D ligand-based pharmacophore modeling is to establish 3D pharmacophores common to active compounds, while matching as few inactives as possible. We proposed a new approach to represent 3D pharmacophores by signatures which will help to identify identical pharmacophores and will be suitable for 3D ligand-based pharmacophore modeling. Pharmacophore is considered to be a complete graph with vertices labeled by pharmacophore feature type and edges representing distances between features in 3D space. However, since stereoisomeric pharmacophores have the same signature, special treatment of stereoconfiguration is required to distinguish pharmacophores with different spatial organization of features. The algorithm is as follows (Figure 1):

1. A particular conformer of a compound is labeled with pharmacophore features using definitions encoded by SMARTS adapted from [14]. A single atom or a fragment can be attributed to several features simultaneously. These features in 3D space represent a 3D pharmacophore. Each feature is attributed to its coordinates, which will be further used for determination of pharmacophore stereoconfiguration.

2. All distances between features are translated to binned distances using a pre-defined step (e.g., 1 Å). Binned distances are used as complete graph edge labels. This will make fuzzy matching possible.

3. All combinations of four features (quadruplets) are considered because four-point objects are the smallest ones, having stereoconfiguration in 3D space. A canonical signature is generated for each quadruplet. The quadruplet signature is a tuple consisting of two parts: one encoding its content and topology (canonical graph signature) and the other encoding stereoconfiguration.

3.1. Content and topology encoding. The quadruplet is considered a complete graph. One round of Morgan-like algorithm [15] is applied to generate canonical identifiers of features taking into account its surroundings. New feature labels consist of the current label of the considered feature and lexicographically sorted labels and binned distances to all other features in a quadruplet (Figure 1). The new feature identifiers are lexicographically sorted, and the obtained tuple represents the canonical graph signature of the pharmacophore quadruplet, which encodes its content and topology.

**Figure 2:**
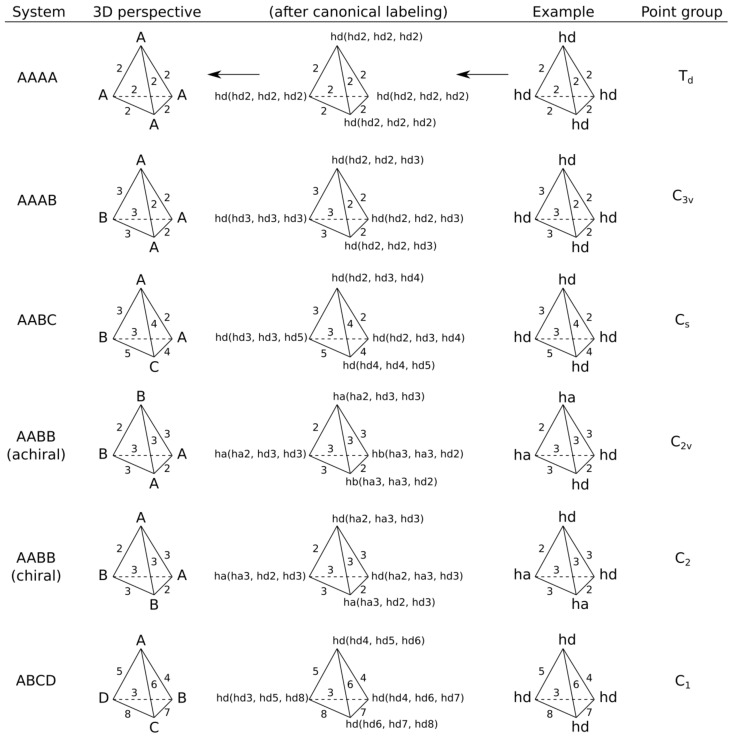
Basic chirality systems. Labels A, B, C and D designate distinct canonical feature labels. Labels “ha” and “hd” designate hydrogen-bond acceptor and donor features, correspondingly. Numbers on edges designate binned distances between features.

3.2. Stereoconfiguration encoding. All quadruplets can be divided into five classes based on the canonical feature identifiers determined in the previous step 3.1 (Figure 2). Capital letters below denote distinct feature labels and do not designate particular feature types.

a) AAAA system, where all features have identical canonical identifiers. This means that four features have identical labels and pairwise binned distances (features create a regular tetrahedron). A quadruplet belonging to this system is achiral.
b) AAAB system, where three features have identical canonical identifiers (A) and one feature has a different one (B). This system corresponds to the trigonal pyramid and is achiral.
c) AABC system, where two features have identical canonical identifiers (A) and two features have different ones (B and C). This system is achiral, because there is a plane of symmetry going through the center of AA distance and B and C features.
d) AABB system, where pairs of features have identical canonical identifiers (A and B). This system can be chiral or achiral depending on distances between pairs of vertices. The achiral one would have a plane of symmetry, whereas the chiral one represents the case of axial chirality.
e) ABCD system, where all features have distinct canonical identifiers. This system is chiral.

**Figure 3:**
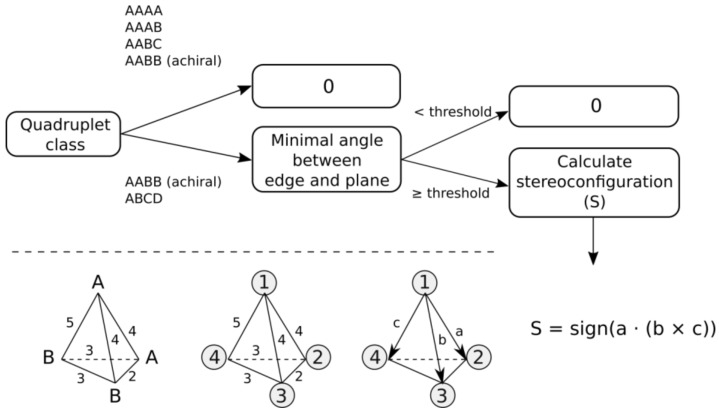
The workflow to determine configuration sign of quadruplets. Labels A and B designate canonical feature identifiers. Numbers on edges designate binned distances. Numbers in circles are ranks of vertices. Labels a, b and c designate vectors.

The general workflow for determining configuration sign is depicted in Figure 3. All quadruplets belonging to AAAA, AAAB or AABC systems are assigned the configuration sign 0. Quadruplets belonging to AABB and ABCD classes can be achiral if all features lie on the same plane. Therefore, first, angles between all edges and corresponding planes are calculated for a quadruplet. The minimum angle characterizes the deviation of a quadruplet from planarity. To tolerate small deviations from planarity, quadruplets having minimal angles smaller than a pre-defined threshold are considered planar and are assigned the configuration sign 0. For the remaining quadruplets, configuration signs (−1 or +1) are assigned based on the sign of the scalar triple product. To calculate the scalar triple product, all vertices are ranked by lexicographic ordering of features based on their canonical identifiers. If two features have the same identifiers (in the case of AABB system), then to break tie, (i) two features having the signature A are placed in a random order, (ii) the feature B having the shortest distance from the first feature A is placed third, and (iii) the remaining feature B is placed fourth (Figure 3). Four ranked features are represented in 3D space by three vectors connecting the top-ranked feature with the remaining ones, as depicted in Figure 3. The scalar triple product is calculated, and its sign determines the configuration of the quadruplet.

**Figure 4:**
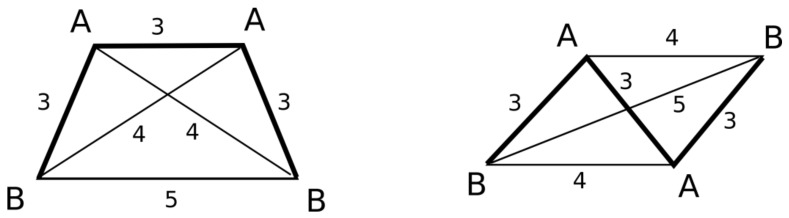
Trapeze-like and parallelogram-like pharmacophore quadruplet belonging to AABB class and not distinguishable by graph canonical signatures.

There are special cases of trapeze-like and parallelogram-like quadruplets belonging to the AABB class that require a specific procedure of stereoconfiguration determination (Figure 4). These types of quadruplets are indistinguishable by the canonical graph signatures calculated in step 3.1 or by the stereoconfiguration signs calculated as described above. Therefore, the calculated stereoconfiguration sign (S) is modified by summing with the signum function of the cosine of the angle B-A-A-B, multiplied by 10.

4. The pharmacophore is considered to be the set of all its quadruplets. The number of quadruplets with identical signatures is calculated. All quadruplets with associated numbers are lexicographically sorted, and the md5 hash of the obtained data structure is calculated (Figure 1). This hash uniquely and compactly encodes the pharmacophore. It is expected that identical hashes will correspond to identical pharmacophores. This might be useful to determine common pharmacophores in ligand-based modeling to derive pharmacophore models. Many different approaches to encode stereoconfiguration were tested, with the one showing the best performance being described above.

### 2.3. 3D Ligand-Based Pharmacophore Modeling

3D ligand-based pharmacophore modeling consists of several stages: (1) training and test set preparation; (2) model development and selection; and (3) external validation of selected models.

#### 2.3.1. Training and Test Set Formation

A data set of active and inactive compounds can be large, and it can be computationally unfeasible to use all available compounds for model development. Therefore, a representative set of active and inactive compounds should be selected for model training.

**Figure 5:**
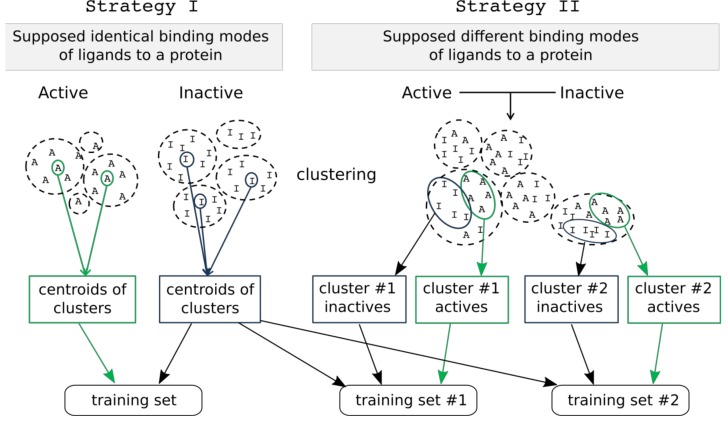
Two strategies of training set compound selection.

Two strategies for training set creation were implemented. The first strategy assumes that all active compounds have the same binding mode, and a representative set is selected from among active and inactive compounds to create a single training set. 2D pharmacophore fingerprints are calculated for all compounds using RDKit. Active and inactive compounds are clustered separately using Butina clustering [16] to find groups (clusters) of similar compounds. Centroids of each cluster of active and inactive compounds having at least 5 compounds are selected for the training set. The number of compounds in the training set depends on the number of clusters which can be tuned by selection of different cutoff thresholds. All remaining compounds form the test set for external validation (Figure 5).

The second strategy assumes that active compounds have different binding modes. In this case, active and inactive compounds are clustered jointly using Butina clustering and 2D pharmacophore fingerprints calculated with RDKit. We assume that compounds from a particular cluster might have similar binding modes relative to other compounds. From each cluster, 5 active and 5 inactive compounds are randomly chosen to form the training set. Clusters containing fewer than 5 active compounds are ignored. Centroids of clusters obtained from clustering of only inactive compounds (similarly to the first strategy) are added to each training set in order to better represent inactive compounds. Duplicated inactive compounds in each training set are removed. Multiple training sets are created using this strategy, and all of them are used for model development. All compounds not included in a particular training set create a complementary test set that is used for external validation of selected models.

As a result, a single training set is created by using strategy I, and multiple training sets by using strategy II.

#### 2.3.2. Model Development and Selection

**Figure 6:**
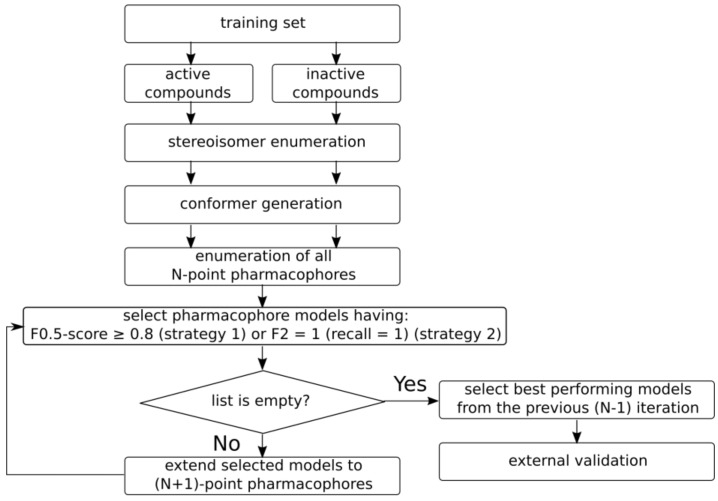
Overall workflow of pharmacophore model generation.

The overall workflow of pharmacophore model generation is depicted in Figure 6. There are two preliminary steps: stereoisomer enumeration, and conformer generation. All stereoisomers are enumerated for molecules having chiral centers and double bonds with undefined stereoconfiguration. The generated stereoisomers are considered to be a single parent compound for the purposes of model development. Up to 100 conformers are generated for each compound/stereoisomer within a 50 kcal/mol range after energy minimization using an MMFF94 force field [17] implemented in RDKit. Such a large energy gap was chosen in order to be able to generate extended structures for highly flexible compounds; otherwise, folded structures would dominate in a conformer set, which may bias the developed models. Thus, each input compound is represented by the set of possible stereoisomers and their conformers.

Pharmacophore model development is an iterative procedure. At the beginning, the 3D pharmacophore hashes of all possible 4-point pharmacophores are calculated for training set compounds. Duplicate hashes obtained for the same compound are dropped out. The occurrence of hashes among active and inactive compounds is calculated, followed by calculation of different statistical characteristics for the training set. Pharmacophores corresponding to hashes which occur mainly in active compounds, rather than in inactive ones, are selected for the next iteration. For a training set formed using strategy I, the pharmacophore models having an F0.5 score greater than or equal to 0.8 are selected. This focuses on the selection of more precise models, rather than those which cover a larger number of active compounds. For a training set formed using strategy II, the pharmacophore models having an F2 score equal to 1 are selected. If there are no such models, then models having a recall equal to 1 are selected. A different criterion for strategy II was chosen because the training sets have a smaller number of active compounds, and it is assumed that these compounds have the same or similar binding modes; therefore, it would be expected to find the model which covers all active compounds from the training set. If the number of models meeting the given criterion is greater than 100, then all pharmacophores having a value of the criterion (F0.5 for strategy I and F2 for strategy II) equal to that of the 100th top model are selected.

On the next iteration, 5-point pharmacophores are generated, adding one feature to the selected 4-point pharmacophores. Hashes and their occurrences are calculated, and the best performing models are selected again for the next iteration. This procedure continues until the generated pharmacophores meet the abovementioned criteria. If there are no pharmacophore models that satisfy the criteria after the current iteration, the models selected on the previous iteration are selected as the final ones, and they are validated on an external test set. The described procedure generates the most complex pharmacophores that match preferably the active compounds in the training set.

Since the same structural patterns can be recognized as different features, e.g., an aromatic feature is always labeled as hydrophobic as well, there will be several features with identical coordinates. Therefore, models with many features may indeed have only a few distinct elements, and the postprocessing step is performed to remove models having three or fewer distinct coordinates of features. These models are considered too simplistic and can be promiscuous.

#### 2.3.3. Database Screening Using Pharmacophore Models

To speed up screening, it is performed in several steps, which include fingerprint screening, isomorphic embedding and hash comparison. In the first step, the hashed fingerprint of a query pharmacophore and dataset molecules’ pharmacophores are generated. Hashed pharmacophore fingerprints are calculated as follows: (i) pharmacophore signatures are calculated for all possible triplets of pharmacophore features using the procedure described above; (ii) these are translated to hashes, which are used as seeds for a pseudo-random number generator; (iii) each triplet activates one bit. The length of a hashed fingerprint bitstring was set at 2048 in this study. Fingerprints are used as a Bloom filter to quickly discard irrelevant pharmacophores: A candidate molecule is relevant for further screening if activated bits in a query pharmacophore fingerprint are the subset of activated bits of the compound.

In the next step, the topology and content of a pharmacophore model are compared with a candidate molecule pharmacophore. A pharmacophore, represented by a complete graph with binned distances, is checked as to whether it is a subgraph (using the VF2 subgraph isomorphism algorithm) of a candidate molecule pharmacophore. In the last step, 3D pharmacophore hashes of a query pharmacophore model and corresponding subgraphs of candidate pharmacophores are compared for identity in order to determine whether they have identical topology and stereoconfiguration.

#### 2.3.4. Model Quality Assessment

Several metrics were used to estimate internal and external performance of models: Recall (true positive rate, TPR), precision, false positive rate (FPR), F-score.

recall (TPR)= TP/P,(1)

precision = TP/(TP + FP),(2)

false positive rate (FPR) = FP/N,(3)

F-score = (1 + β^2)∙(precision∙recall)/(β^2∙precision + recall)(4)

where P is the total number of active compounds in a data set, N is the total number of inactive compounds in a data set, true positives (TP) is the number of correctly predicted active compounds, false positives (FP) is the number of inactive compounds predicted as actives, β is a factor giving more weight to precision or recall when F-score is calculated (0.5 or 2 in this study).

## 3. Results and Discussion

The influence of clustering cutoff (0.3, 0.4 and 0.5) and tolerance threshold (0, 5 and 10) on performance of generated models was studied. The former enables controlling of the number of clusters and, subsequently, training set size and the number of training sets. The greater the cutoff value is, the smaller number of clusters will be. Correspondingly, there will be a smaller number of training set compounds in strategy I and a smaller number of training sets in strategy II (Table 2).

Higher tolerance threshold values should help ignore small deviations in quadruplet planarity. This may result in a greater number of pharmacophore matches during training and validation. However, in this study, we did not observe such dependence. Results obtained with tolerance thresholds of 5 and 10 were rarely substantially different from those obtained at tolerance 0. Therefore, only the results obtained with 0 tolerance will be discussed in detail below, with all results being provided in the Supplementary materials.

The number of obtained pharmacophore models was variable for different clustering cutoff values, whereas model complexity was almost the same. The complexity (number of features) of models obtained from the training sets created by strategy I was lower than the complexity of models trained on compound subsets selected by strategy II (Table 3). This is to be expected, since the compounds selected by strategy I are more diverse and less likely to have a large number of common features.

**Figure 7:**
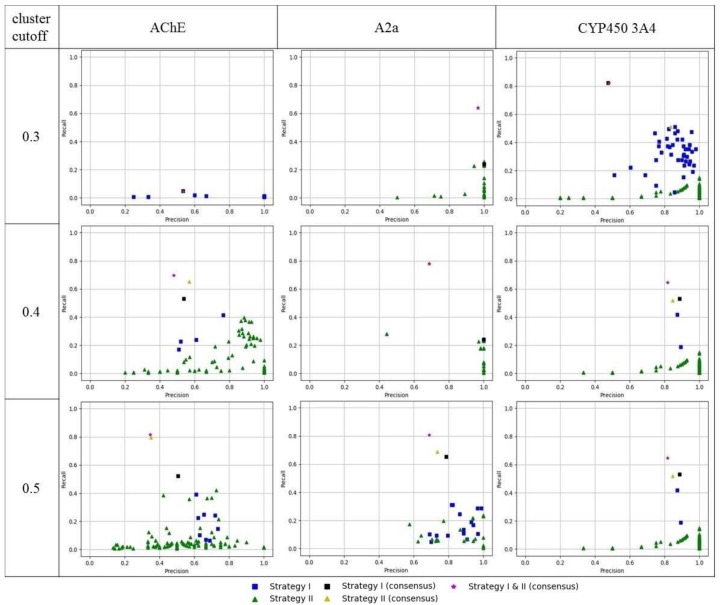
Precision and recall of obtained 3D ligand-based pharmacophore models.

External validation of the obtained 3D pharmacophore models was performed on a subset of compounds which were not included in the training set of a particular model. As expected, models obtained by strategy I mainly had higher recall values but lower precision relatively than models obtained by strategy II (Figure 7). Therefore, models obtained by strategy II can be recommended for conservative predictions where there is a need to retrieve true hits with high probability. Whereas models obtained by strategy I are more risky, but can retrieve a more diverse list of hits with lower chances of finding true hits.

Consensus predictions were made by combining the retrieved lists of hits from individual models and removing duplicate entries. Recall of consensus models was better than that of individual models. Precision of consensus models was more sensitive than precision of individual models. Inclusion of less precise models greatly reduces the precision of the consensus predictions, but it was usually at a reasonable level. Therefore, consensus models can be recommended for virtual screening, because they provide a good trade-off between precision and the number of retrieved true hits (recall).

**Figure 8:**
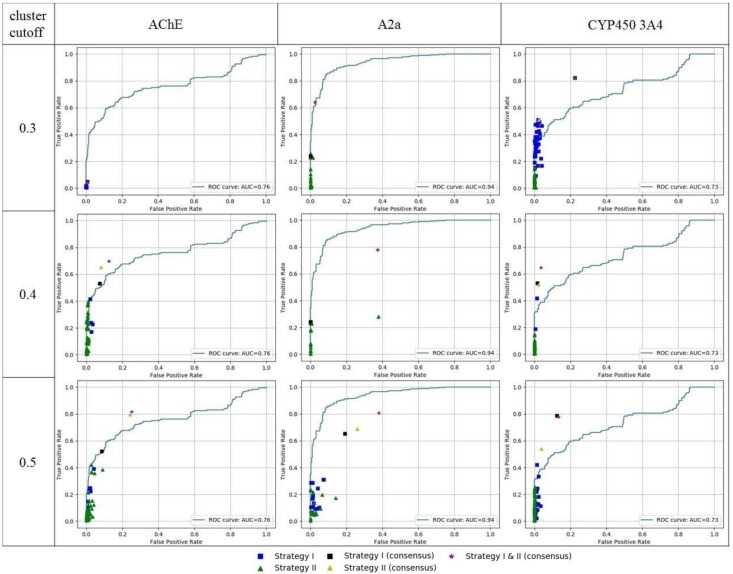
Performance of 3D ligand-based pharmacophore models and comparison with the best results of similarity search based on 2D pharmacophore fingerprints.

The obtained results of 3D ligand-based pharmacophore modeling were compared with a 2D similarity search based on RDKit pharmacophore fingerprints. Each active compound from a whole data set was used as a reference, and all remaining compounds were ranked according to Tanimoto similarity to that reference in order to build a ROC curve and calculate the area under curve (AUC) value. The compound with the highest AUC value was selected from each data set for comparison with the output of the pharmacophore models. This is the most rigorous comparison scenario. Pharmacophore model performance was expressed as TPR and FPR in order to compare with similarity search results (Figure 8).

In many cases, model performance was very close or identical to the best similarity search results. In some cases, e.g., for the CYP450 3A4 data set, performance of models obtained with strategy I was better than the similarity search (points are above ROC, Figure 8). However, models obtained with strategy II had a performance similar to the best similarity search results (points lie on a ROC) but did not outperform them. This can be explained by the fact that these models (strategy II) were trained on more congeneric subsets of compounds, representing a smaller subspace of available ligands relative to models trained on a diverse subset of compounds (strategy I). Consensus models outperformed the similarity search results for the CYP450 3A4 and AChE data sets.

**Figure 9:**
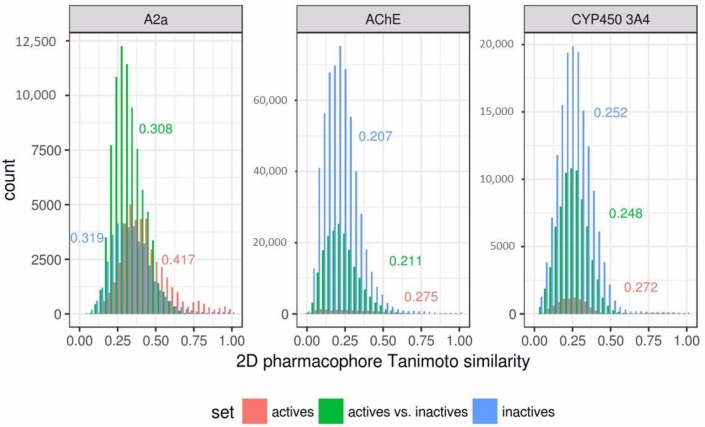
Distribution of pairwise Tanimoto similarity calculated based on 2D pharmacophore fingerprints for subsets of active and inactive compounds of each data set. Numbers are median pairwise Tanimoto values for each subset.

The relatively poor performance of the 3D pharmacophore models in comparison to the similarity search results obtained for the antagonists of adenosine 2a receptor can be explained by a bias in the data set. This data set was balanced (Table 1). The active compounds in this data set are much more similar than the inactives, with respect to the other two data sets (Figure 9). It seems that it contains a large number of congeneric compounds. This conclusion is supported by the low total number of obtained clusters (Table 2). Active compounds were concentrated in only several clusters. Even at the highest selected cutoff (0.5), there were three large clusters highly populated with active compounds: cluster 1 (145 actives out of 204 compounds), cluster 2 (61 actives out of 61 compounds), and cluster 3 (49 actives out of 49 compounds). These three clusters contain 255 of a total of 293 active compounds from the data set. Due to such a high homogeneity, the similarity search performed extremely well, and the AUC achieved a value of 0.96 in the best case, which outperforms the obtained 3D pharmacophore models. 3D pharmacophore models trained with the second strategy were accurate, but could not retrieve a large number of actives, because these models were trained on separate clusters and matched particular features present in compounds of those clusters. Consensus prediction of these models substantially improved the number of retrieved active compounds (TPR); this strategy performs especially well in the case with cluster cutoff of 0.3. In other cases, due to several poor individual models which retrieved a lot of inactives, the consensus prediction also retrieved many false positives. 3D pharmacophore models trained with the first strategy were also less accurate than the similarity search, because they were trained on compounds that were centroids of clusters. The training set contained only one compound from each of mentioned three clusters highly populated with active compounds. Therefore, compounds of these clusters were underrepresented in the training set. It seems that it would be more reasonable to select compounds proportionally to cluster sizes.

**Figure 10:**
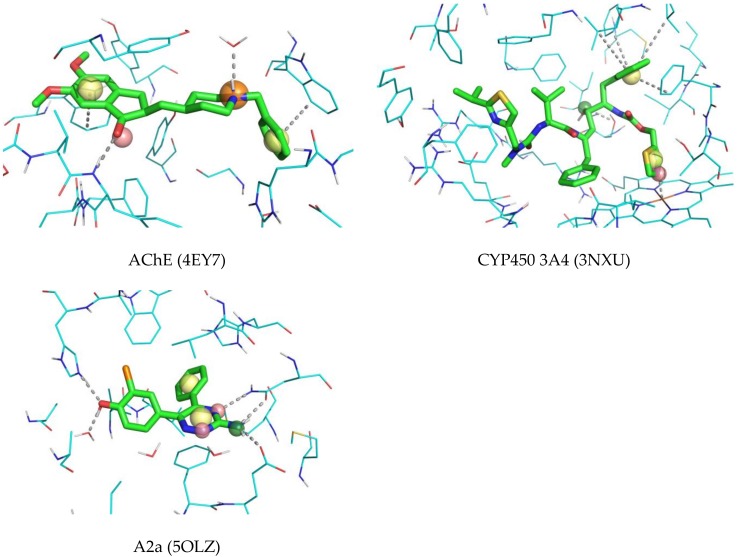
Examples of compounds matching corresponding 3D pharmacophore models developed in this study. Red spheres denote H-bond acceptors, yellow spheres—hydrophobic/aromatic features, orange spheres—H-bond donor and positively charged centers, green sphere—H-bond donor and H-bond acceptor features.

The obtained 3D ligand-based pharmacophores were used to screen 3D poses of available ligands taken from ligand-protein PDB complexes. In total, 9 antagonists of adenosine 2a receptors, 10 inhibitors of AChE and 26 inhibitors of CYP450 3A4 which were not present in the corresponding sets used to train the models were collected from PDB. 5 antagonists of adenosine 2a receptors, 2 inhibitors of AChE, and 3 inhibitors of CYP540 3A4 were found by at least one model. Donepezil, an AChE inhibitor, fits the 3D pharmacophore model that contains features corresponding to observed ligand-protein interactions (Figure 10). Two phenyl rings are involved in interaction with Trp86 and Trp186, and the carbonyl oxygen forms an H-bond with the backbone of the Phe295 residue. The hydrophobic feature of one of the CYP450 3A4 pharmacophore models matches the phenyl group of ritonavir, which fits the hydrophobic pocket of the protein formed mainly by phenylalanine and leucine residues (Figure 10). Another hydrophobic feature and H-bond acceptor match the thiazolyl ring which coordinates the heme. The hydroxyl group matching H-bond donor and acceptor features forms H-bond with the Ser119 side chain. The A2a pharmacophore model matched most of observed ligand-protein interactions for the ligand in the 5OLZ complex (Figure 10). The H-bond donor matches the amino group, which forms H-bonds with Glu169 and Asn253. H-bond acceptor features match two nitrogen ring atoms. One of them forms an H-bond with Asn253 and another one with Glu159 through a water molecule. Phenyl group matching a hydrophobic feature of the pharmacophore is in the pocket close to Met177, Trp246 and Leu249. These examples demonstrate agreement between the 3D ligand-based pharmacophore models developed within the proposed approach and the actually observed 3D poses of corresponding ligands in their complexes.

## 4. Conclusions

A new approach to 3D pharmacophore representation was developed and successfully tested in several retrospective cases. The developed 3D pharmacophore signatures can be used for ligand-based pharmacophore modeling that does not require pre-defined geometry of active compounds to be used as a template, or the explicit alignment of pharmacophores.

Two strategies of training set selection were implemented. One is based on an assumption that all active compounds have a similar binding mode; the other assumes that they have different binding modes. Models obtained with strategy II were more accurate in their predictions, but retrieved a lower number of actives than strategy I models, because the former were more complex and covered specific subspace of active compounds. However, both of them can be useful. Strategy II models can give more conservative but more confident predictions, whereas strategy I models can result in a large number of diverse compounds. Consensus predictions made by combining predictions of individual models outperformed those of individual models and retrieved an even greater number of true hits, whereas their precision was lower than the precision of the individual models. Therefore, the use of consensus predictions can be recommended if the goal is to retrieve a large number of diverse hits.

Performance of the developed 3D pharmacophore models was comparable to or higher than the performance of a 2D pharmacophore similarity search. The developed 3D ligand-based pharmacophores were able to match important ligand-protein contacts of known ligands in their observed poses taken from PDB protein-ligand complexes. These prove the validity and applicability of the developed approach to retrieve reasonable 3D ligand-based pharmacophores. The developed approach does not allow the creation of exclusion volumes to restrict the shape and size of matched compounds. However, the current implementation shows good performance on external validation.

The developed tool is open-source software, whereas the available free tool for ligand-based pharmacophore modeling (PharmaGist) is web-based. They are easy to use, but their availability depends on the funding of a corresponding research group. The developed standalone application does not have such issues.

## Figures and Tables

**Table 1:** Data sets used for ligand-based pharmacophore modeling.

Data Set	Number of Actives	Number of Inactives	Total Number of Compounds
AChE	176 (pIC_50_ ≥ 8)	1070 (pIC_50_ ≤ 6)	1246
CYP450 3A4	138 (pIC_50_ ≥ 7)	548 (pIC_50_ ≤ 5)	686
A2a	293 (pKi/pKd/pIC_50_ ≥ 7)	279 (pKi/pKd/pIC_50_ ≤ 5)	574

**Table 2:** Results of data set clustering and training sets size and number.

Data Set/Cluster Cutoff	Total Number of Clusters	Number of Active/Inactive Compounds in the Training Set (Strategy I)	Number of Training Sets (Strategy II)
AChE / 0.3	393	12/60	9
AChE / 0.4	280	12/62	11
AChE / 0.5	197	7/52	7
A2a / 0.3	139	13/13	12
A2a / 0.4	95	11/12	10
A2a / 0.5	59	6/14	5
CYP 3A4 / 0.3	293	8/23	7
CYP 3A4 / 0.4	233	7/27	7
CYP 3A4 / 0.5	154	8/27	7

**Table 3:** Complexity (number of features) of pharmacophore models.

	AChE	A2a	CYP450 3A4
strategy I	5	6–8	6
strategy II	5–9	5–10	7–9

## Supplementary Materials

The following are available online at http://www.mdpi.com/1420-3049/23/12/3094/s1, Figure S1: Precision-recall plot of 3D ligand-based pharmacophore models built on AChE inhibitors, Figure S2: Precision-recall plot of 3D ligand-based pharmacophore models built on adenosine 2a antagonists, Figure S3: Precision-recall plot of 3D ligand-based pharmacophore models built on CYP450 3A4 inhibitors, Figure S4: Performance of 3D ligand-based pharmacophore models built on AChE inhibitors and comparison with the best result of similarity search based on 2D pharmacophore fingerprints, Figure S5: Performance of 3D ligand-based pharmacophore models built on adenosine 2a antagonists and comparison with the best result of similarity search based on 2D pharmacophore fingerprints, Figure S6: Performance of 3D ligand-based pharmacophore models built on CYP450 3A4 inhibitors and comparison with the best result of similarity search based on 2D pharmacophore fingerprints.
