# Correction: Kutlushina, A., et al. Ligand-Based Pharmacophore Modeling Using Novel 3D Pharmacophore Signatures. *Molecules*, 2018, *23*, 3094

**DOI:** 10.3390/molecules24061052

**Published:** 2019-03-18

**Authors:** Alina Kutlushina, Aigul Khakimova, Timur Madzhidov, Pavel Polishchuk

**Affiliations:** 1 A.M. Butlerov Institute of Chemistry, Kazan Federal University, Kremlevskaya Str. 18, 420008 Kazan, Russia; Alina.Kutlushina@pharminnotech.com (A.K.); aigul03.14@gmail.com (A.K.); tmadzhidov@gmail.com (T.M.); 2 Institute of Molecular and Translational Medicine, Faculty of Medicine and Dentistry, Palacký University and University Hospital in Olomouc, Hnevotinska 5, 77900 Olomouc, Czech Republic

The authors would like to add the funding number to the published article [1]. The corrected Funding section is provided below:

**Funding:** This research was funded by the Ministry of Education, Youth and Sports of the Czech Republic, agreement number MSMT-5727/2018-2 and by the Ministry of Education and Science of the Russian Federation, agreement No14.587.21.0049 (unique identifier RFMEFI58718X0049).

The manuscript will be updated, and the original will remain online on the article’s webpage. The authors would like to apologize for any inconvenience caused to the readers by these changes.
