# Supplementary material for: Ligand-Based Pharmacophore Modeling Using Novel 3D Pharmacophore Signatures
Source: Molecules. 2018 Nov 27;23(12):3094. doi: 10.3390/molecules23123094 (PMC6321403; doi:10.3390/molecules23123094)
Supplement: Supplementary file 1 [file molecules-23-03094-s001.zip › si.pdf]

Ligand-based pharmacophore modeling using novel 3D pharmacophore signatures

Alina Kutlushina [1], Aigul Khakimova [1], Timur Madzhidov [1], Pavel Polishchuk [2,*]

[1] A.M. Butlerov Institute of Chemistry, Kazan Federal University, Kremlevskaya Str. 18, Kazan, Russia
[2] Institute of Molecular and Translational Medicine, Faculty of Medicine and Dentistry, Palacký University and University Hospital in Olomouc, Hnevotinska 5, 77900 Olomouc, Czech Republic
* Correspondence: pavlo.polishchuk@upol.cz; Tel.: +420 585632298

Supporting materials

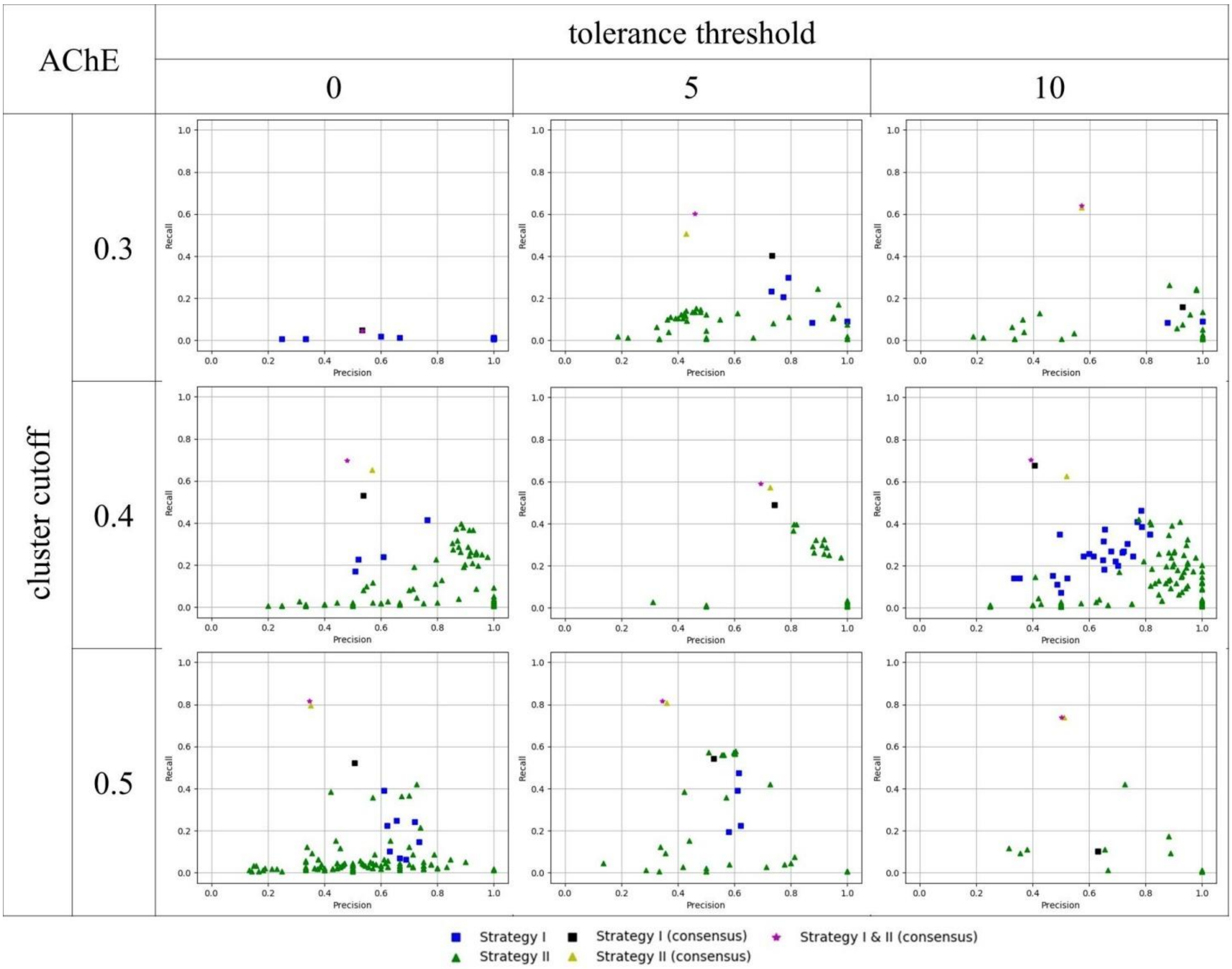

Figure S1. Precision-recall plot of 3D ligand-based pharmacophore models built on AChE inhibitors.

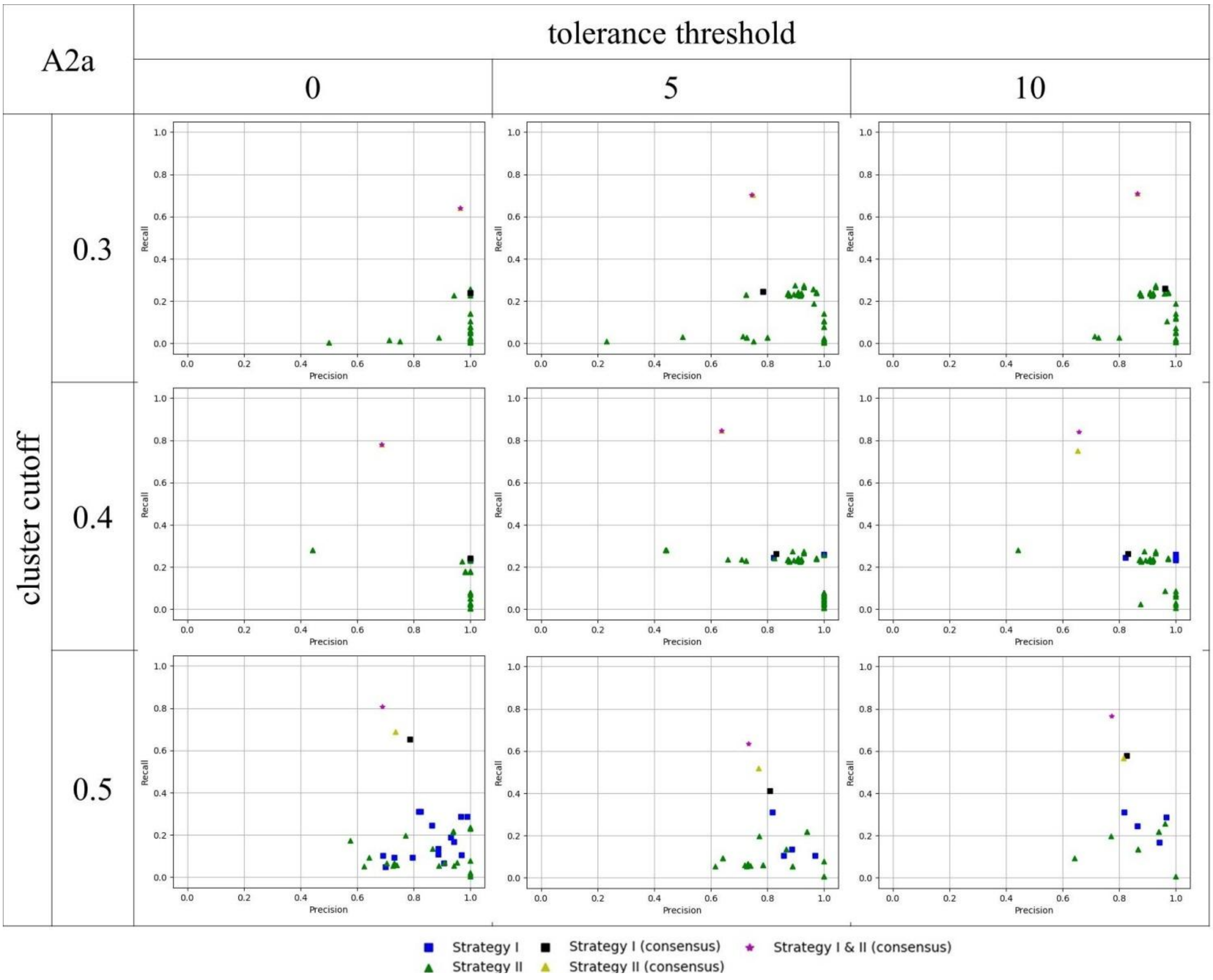

Figure S2. Precision-recall plot of 3D ligand-based pharmacophore models built on adenosine 2a antagonists.

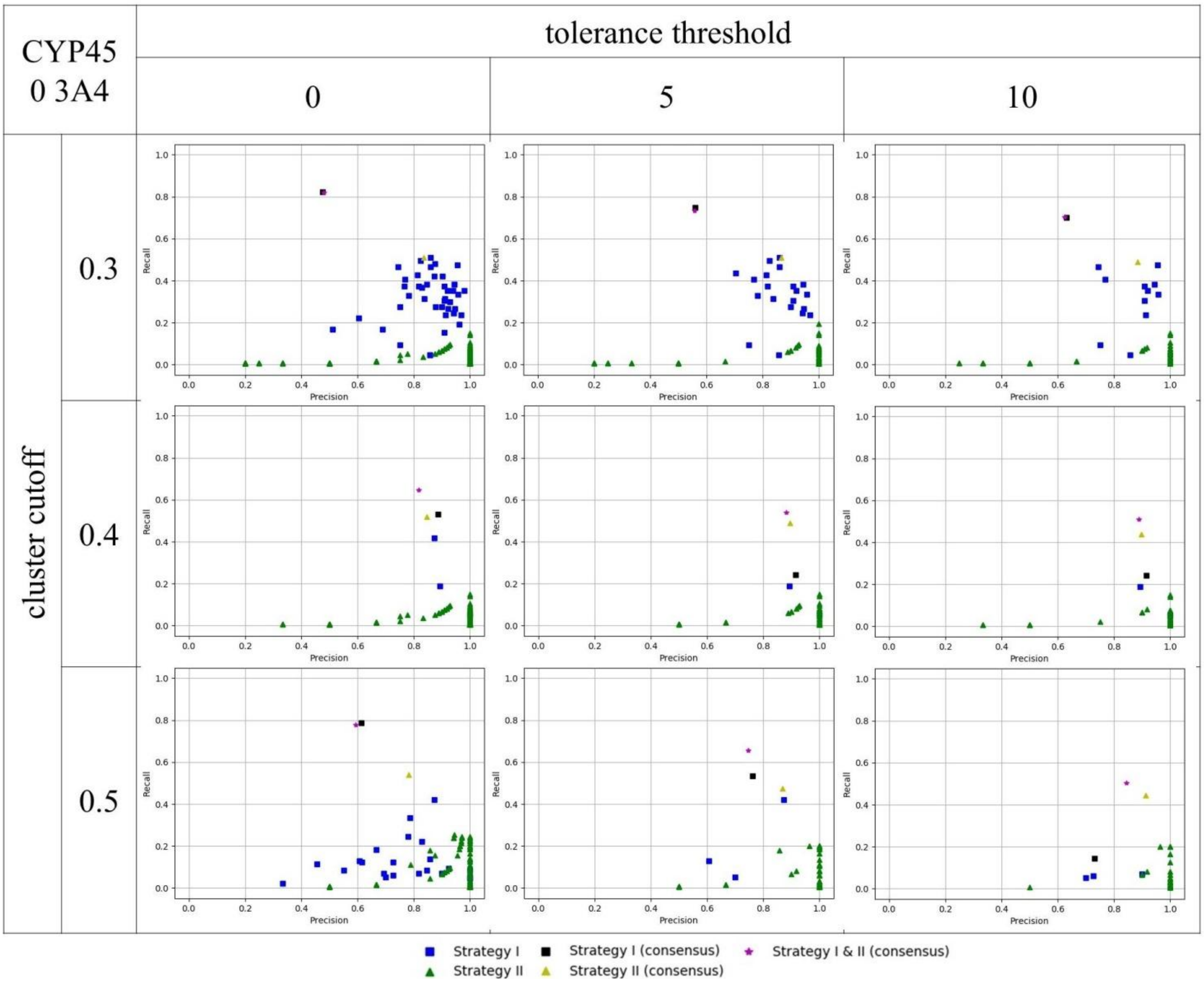

Figure S3. Precision-recall plot of 3D ligand-based pharmacophore models built on CYP450 3A4 inhibitors.

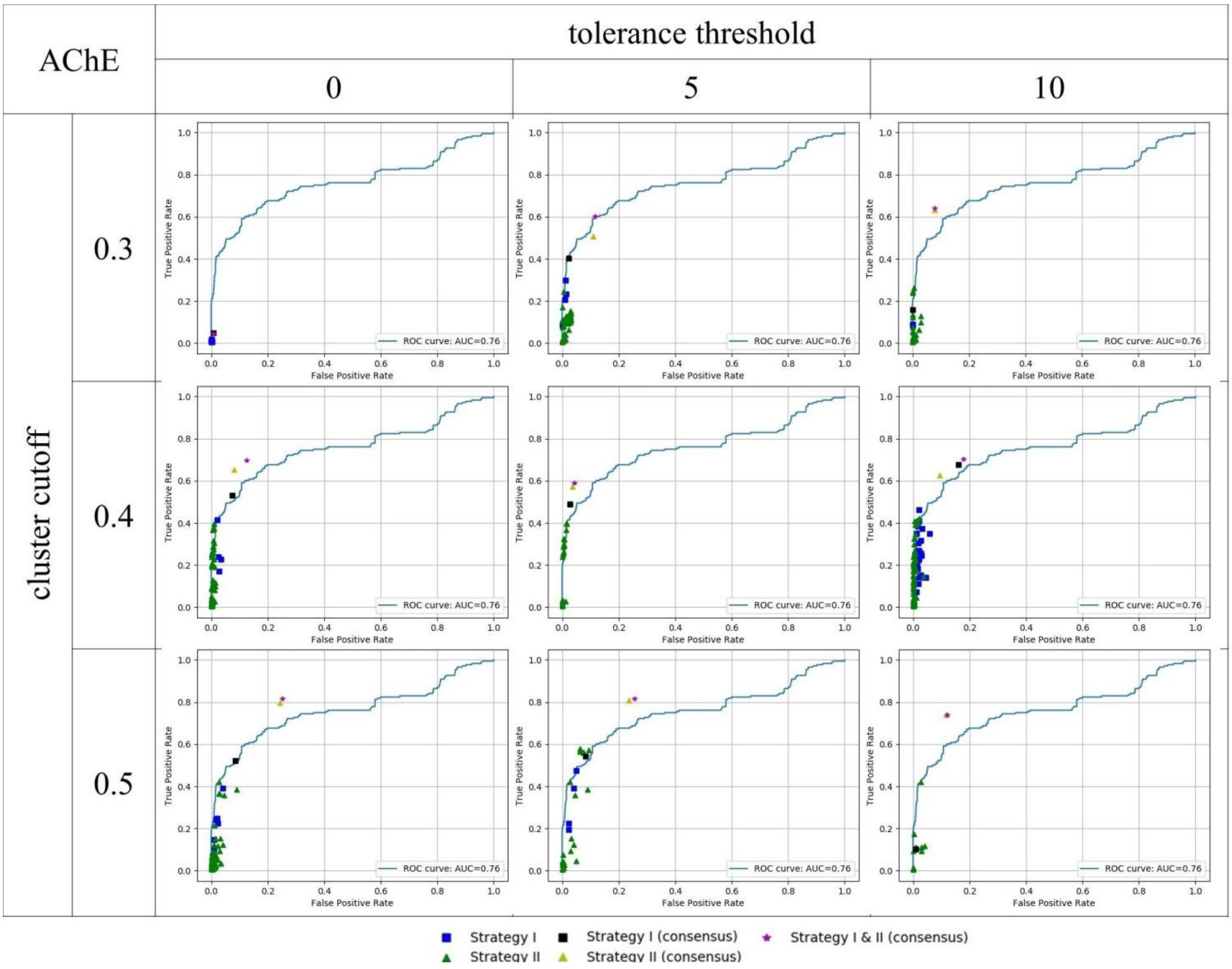

Figure S4. Performance of 3D ligand-based pharmacophore models built on AChE inhibitors and comparison with the best result of similarity search based on 2D pharmacophore fingerprints.

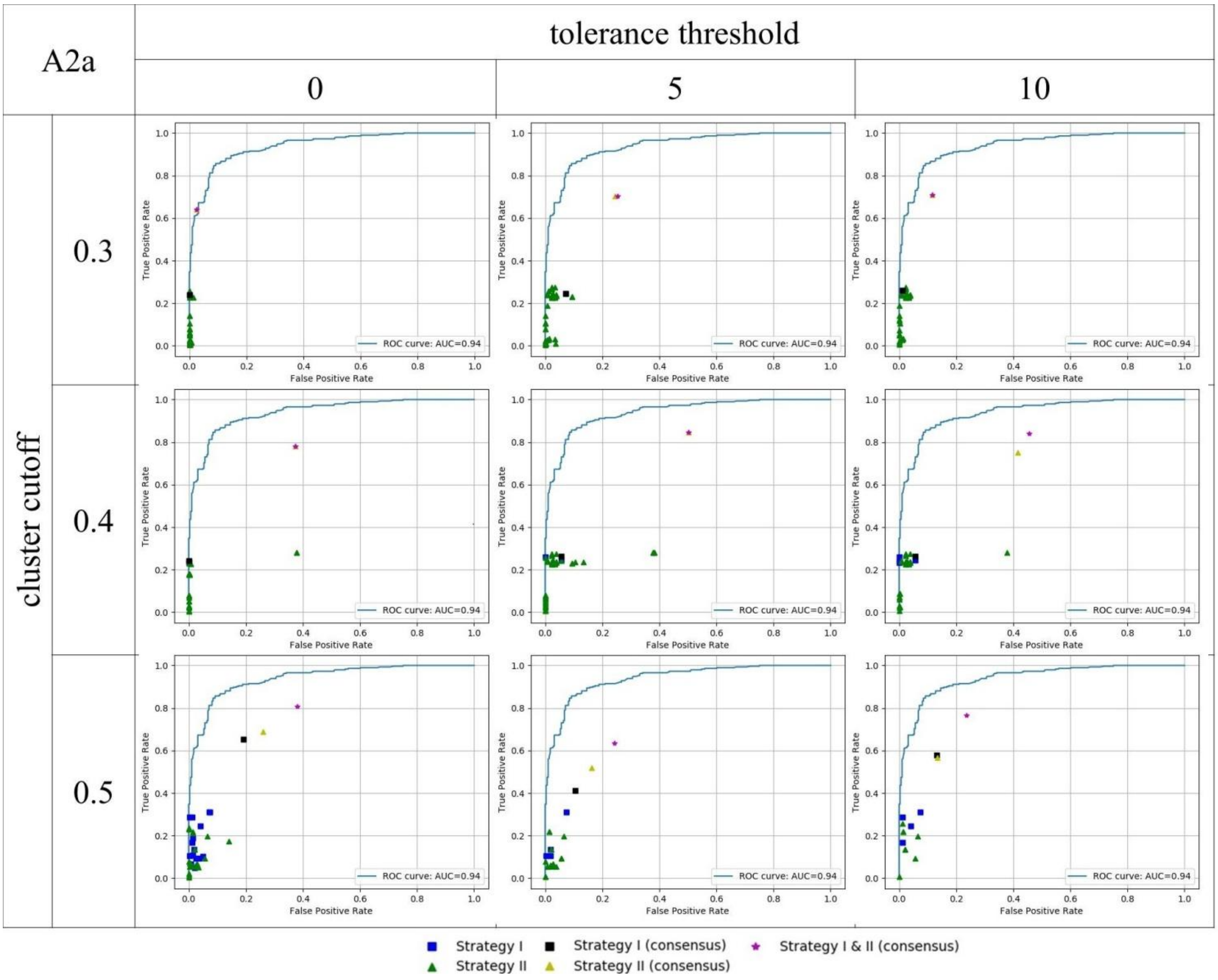

Figure S5. Performance of 3D ligand-based pharmacophore models built on adenosine 2a antagonists and comparison with the best result of similarity search based on 2D pharmacophore fingerprints.

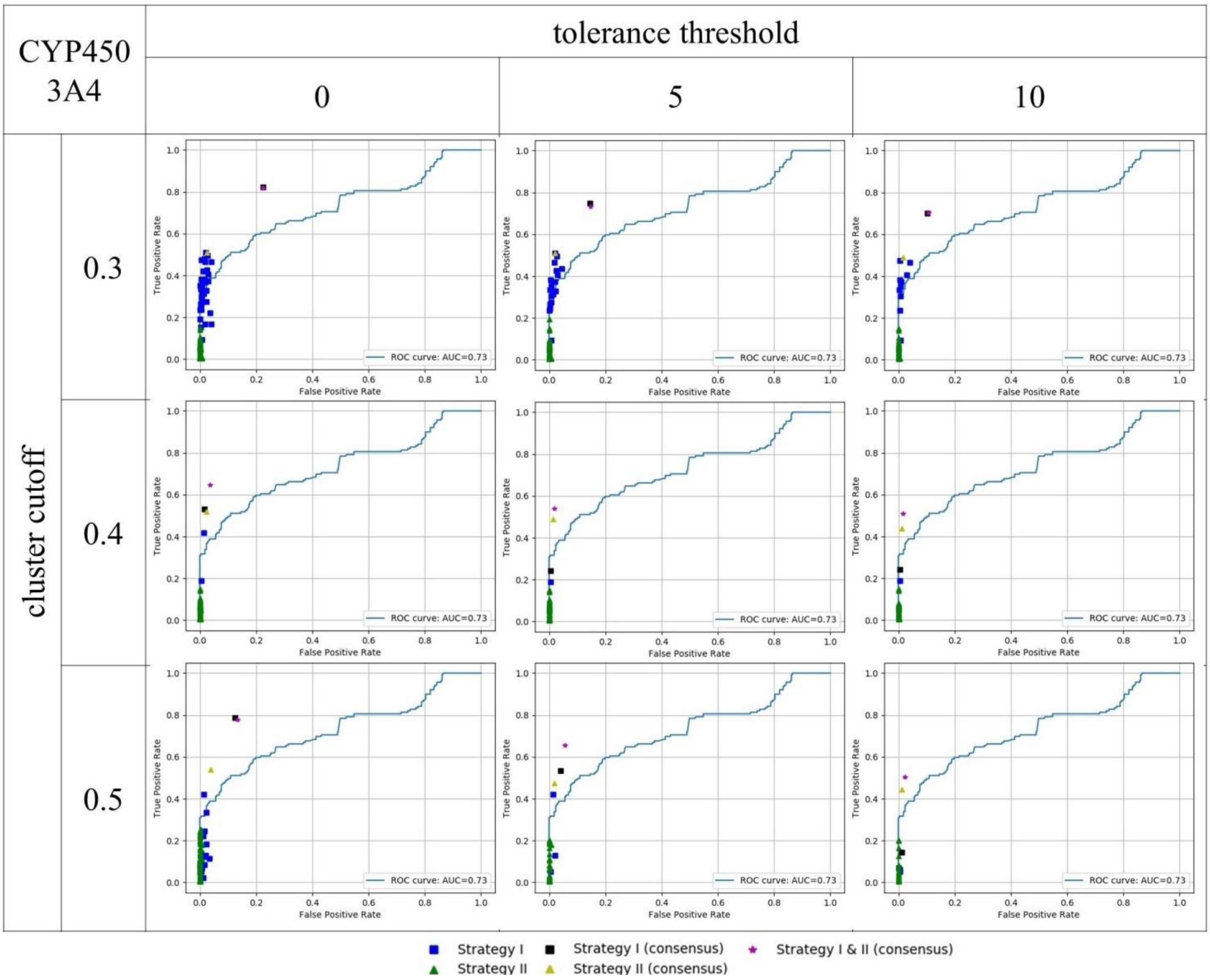

Figure S6. Performance of 3D ligand-based pharmacophore models built on CYP450 3A4 inhibitors and comparison with the best result of similarity search based on 2D pharmacophore fingerprints.
